# The European Research Council—A European Renaissance

**DOI:** 10.1371/journal.pbio.0020161

**Published:** 2004-05-11

**Authors:** Bill O'Neill

## Abstract

European scientists are pressing for the creation of an independent body to fund European research - driven by the pursuit of scientific excellence

Science looks set for a fundamentalist revival within the European Union. Its leading proponents are taking advantage of unprecedented political upheaval—as ten new Member States accede to the Union—to press their case for funding of basic research that is driven solely and independently by investigators themselves in the pursuit of excellence.[Fig pbio-0020161-g001]

**Figure pbio-0020161-g001:**
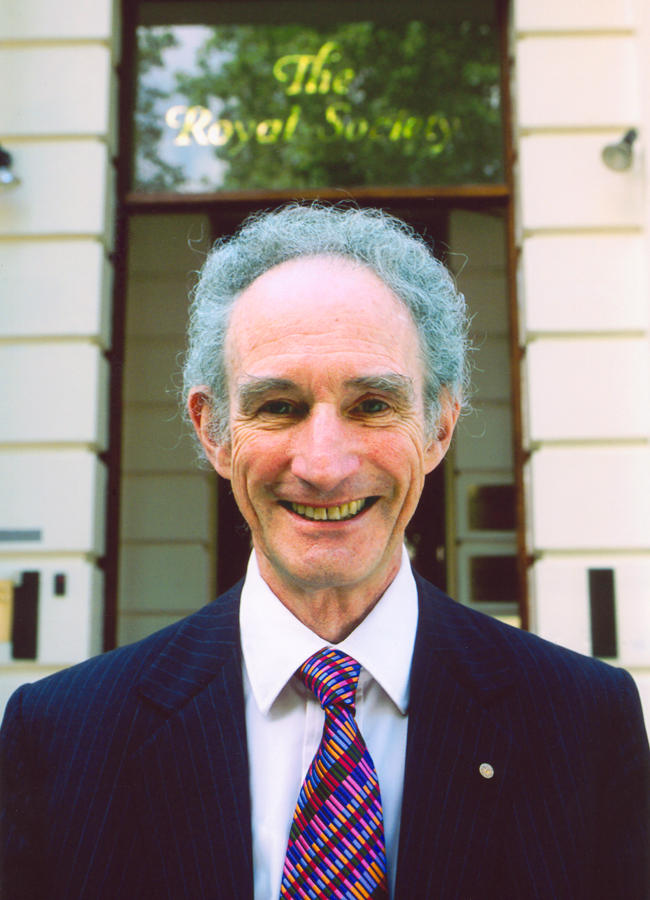
Bob May, professor of mathematical biology at the University of Oxford, president of the Royal Society, and former UK Chief Scientist

The broad thrust of their appeal calls for the setting up of a new agency, most commonly referred to as a ‘European Research Council’. The ERC could be an entirely new organisation or a new division within an established body, run by a small staff able to draw on the best expertise available. It would administer a new fund from EU coffers, tagged the European Fund for Research Excellence, that would be valued modestly, initially at least, at much less than half of the EU's existing budget for research. Most importantly, dispersal of that fund would reflect the wishes of eminent peer reviewers, assessing competitive bids in search of the best science, rather than the judgements of Eurocrats, looking for the most politically and economically expedient solutions and operating on a lead time of two years or more.

Although the modus operandi of the proposed ERC has still to be worked out, European scientists have been looking to the United States and at the way that the National Science Foundation and the National Institutes of Health operate, as well as to private institutions such as the Howard Hughes Medical Institute in the United States and the Wellcome Trust in the United Kingdom. In particular, they seek the independence and excellence achieved outside of the EU framework. More to the point, they are weary of the bureaucratic formulations that determine how the EU's research budget, currently known as the Sixth Framework Programme (2002–2006) and worth around €4.4 billion/year (or just over 5% of all public spending on nonmilitary research in the region), is spent and distributed. The EU's guiding principle is often one of *juste retour*, or fair reward, in which Member States traditionally seek to recover grants at least equal to their contributions to the EU pot (see Box 1).[Fig pbio-0020161-g002]

**Figure pbio-0020161-g002:**
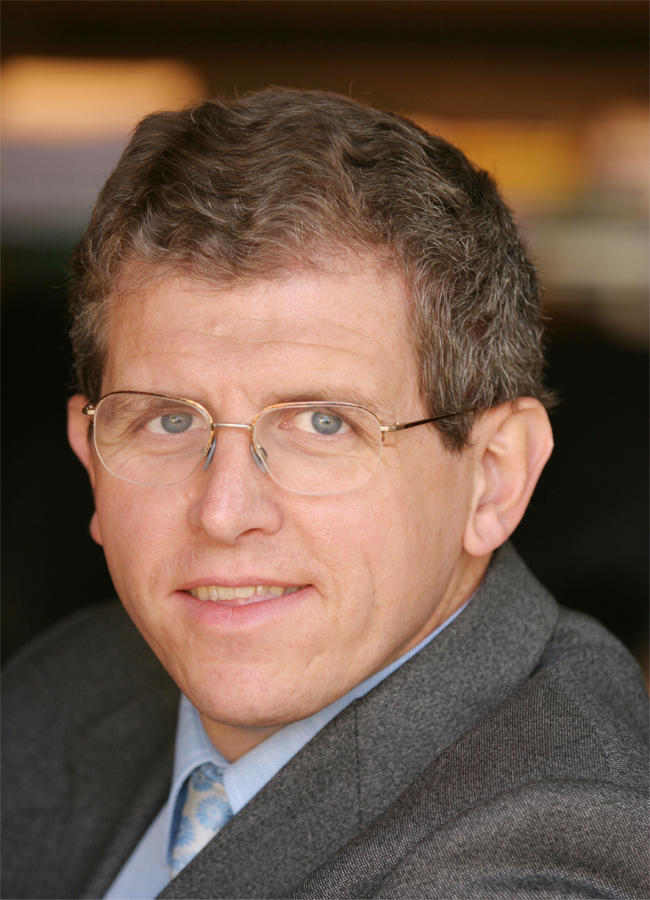
Bernard Larrouturou, director general of France's National Centre of Scientific Research (CNRS) in Paris

‘Most of the Anglo-Saxon countries in Europe—the Scandinavian countries, the United Kingdom, the Netherlands—operate a peer review process and a research funding council process that's very similar to best practice in North America,’ says Michael Morgan, a consultant to the Wellcome Trust on European issues and former chief executive of the Trust's Genome Campus at Hinxton, near Cambridge, United Kingdom. ‘The French and Germans and others have elements of that but they also have what you might call more “state-funded science”, scientists as civil servants, and there is obviously much greater possibility of science being funded for less than the best scientific reasons,’ notes Morgan, referring to the opportunities for greater political influence on decision-making. ‘I'm not suggesting that that is the case, but it is the possibility,’ he adds.

‘What we need in Europe is something that should strictly adhere to the international standards of research funding and be evaluated by peer review,’ says Peter Gruss, professor of molecular cell biology at the University of Göttingen and president of the Max Planck Society in Munich, Germany. ‘The sole criterion has to be quality, not geographical distribution, not management capacity,’ he adds, alluding to the EU practice of *juste retour*. ‘We want to encourage excellence in Europe. We want to have as a benchmark a European standard that should be as high as the standard is in the US.’[Fig pbio-0020161-g003]

**Figure pbio-0020161-g003:**
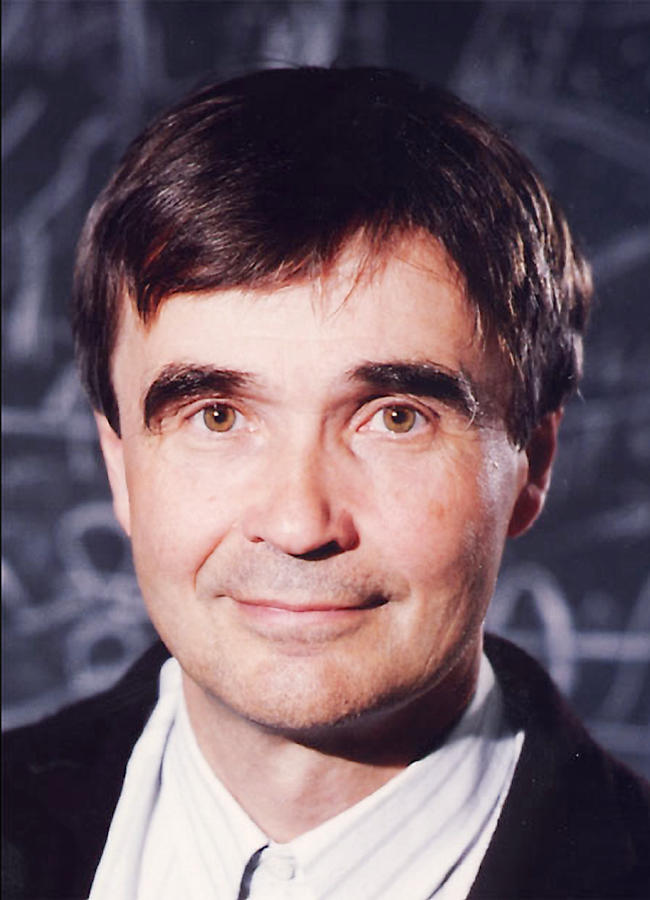
Kai Simons, president of the ELSO and director of the Max Planck Institute for Cell Biology and Genetics

Gruss acknowledges the tensions that the ERC proposal has generated among Member States: ‘I'm not saying that there aren't countries that have this standard—like the UK, parts of Germany, Sweden, and some other Nordic countries—but of course this is not the general European standard, and in order to get one and the same, the common standard, we need a common structure.’

## A Fund for Excellence

The European Commission now appears ready to accept the need for a common structure that would have, as the Commission puts it, ‘more open and less binding’ programmes of basic research, in contrast to the Framework Programme, whose emphasis is on applied research with commercial objectives. The Commission expects to publish its endorsement of the ERC proposal this month, so that approval by the Council of the EU should follow later this year. On this timetable, setting up of the ERC could begin in 2006 when the next five-year Framework Programme, FP7, gets underway.

Over the ERC's first five years, its grant is expected to grow from around €500 million/year to €2 billion/year, and to derive from a reallocation of funds within the EU's budget rather than from any top-up contributions from Member States. Furthermore, Gruss released a legal opinion in March that advised how an ERC need not be an executive agency of the Commission, as many scientists had feared it would have to be under the terms of the EU Treaty, but could be established as an independent and autonomous body. The opinion is a real coup for the ERC lobbyists.

## Origins of the ERC

Moves to establish an ERC are founded in a ‘new strategic goal’ for the EU that the leaders of its 15 Member States set during their European Council in Lisbon in March 2000. Over the first decade of the new millennium, they urged the EU ‘to become the most competitive and dynamic knowledge-based economy in the world’. They enthusiastically endorsed a notion, floated by the European Commission, of a European Research Area (ERA). ‘Research activities at national and [European] Union level must be better integrated and co-ordinated to make them as efficient and innovative as possible, and to ensure that Europe offers attractive prospects to its best brains,’ concluded the EU leaders, eager to reverse the flow of trained talent abroad, notably to North America. All appropriate means, they added, ‘must be fully exploited to achieve this objective in a flexible, decentralised and non-bureaucratic manner’.

Two years later, at the European Council in Barcelona, the EU leaders went one step further by defining the target more precisely. ‘In order to close the gap between the EU and its major competitors,’ they said, ‘overall spending on R & D and innovation in the Union should be increased with the aim of approaching 3% of GDP by 2010. Two-thirds of this new investment should come from the private sector.’

The scale of the challenge is illustrated by the latest figures for R & D expenditure, published in February by the Statistical Office of the European Communities (Eurostat). The EU's estimated R & D spending in 2002 was 1.99% of GDP, still far short of the US (2.80%) and Japan (2.98% in 2000), and a long way from the target of 3%.

Emphasising the UK's uneasiness about the EU's escalating enthusiasm for a regional science base, the Royal Society (the UK national academy of science) poured scorn on the ‘ambitious’ GDP target by noting how the UK alone would have needed an extra £11 billion in 2000, or more than 60% of total spending on R & D, to lift its ratio of 1.85% to the 3% target. The Royal Society also noted how public funding of R & D in the EU matches that in the US and Japan, with the disparity among GDP ratios reflecting the differentials in private investment in R & D, over which the EU has little control.

Nevertheless, the challenge could not be ignored. According to Bob May, professor of mathematical biology at the University of Oxford, president of the Royal Society, and former UK Chief Scientist, such initiatives might be ‘driven more by political expediency than common sense, but the moment you see that train beginning to roll, there's a chance to do something useful with it’.

Among the leading proponents of an ERC is Bernard Larrouturou, director general of the National Centre of Scientific Research (CNRS) in Paris, France. For Larrouturou, a biomathematician currently engaged in streamlining the organisation, the changes at the European level are a breath of fresh air. However, he is not convinced that funded investigators should expect to exclude Commission strategists entirely from their lives. The scientific community should lead an ERC, says Larrouturou, ‘but I do not like the idea that this should be completely under the guidance and wisdom of the scientific community with no strategy guidance. You cannot ask for 1 or 2 billion Euros every year and say there will not be any strategy and [that it will be done solely] on this basis of excellence.’ And Larrouturou distances himself from the idea that basic and applied research can be treated separately because this suggests, wrongly he says, a conflict between the two.

On these issues, Larrouturou moves onto some common ground with John Taylor, former director general of Research Councils UK and now chairman of Roke Manor Research, a UK subsidiary of Siemens, the German electronics group. Research Councils UK oversees spending of Britain's national research councils (currently, just over £2 billion from its 2004–2005 Science Budget of nearly £2.7 billion). Interactions across disciplines and between scientists and technologists ‘are not helped by making artificial distinctions between this kind of research and that kind of research,’ says Taylor. ‘The distinctions I make are much more between top-down and bottom-up.’

While Taylor is a joint architect of one proposal to create an ERC, he remains unconvinced that the research funding system is broken, especially from the UK's perspective, and needs to be fixed. Nor is he convinced that EU funds for an ERC will not affect national R & D budgets. ‘I'm middle of the road,’ he says. ‘Much greater collaboration is good. It has to be a slow process, with all the different cultures involved. Collaboration on various areas of science is an excellent way to go, provided you don't try to organise it from the top and legislate for it all to happen in a particular way and to a particular timescale. Excellence is key.’

Taylor's cautions reflect his experience of the EU's Framework Programme and his reservations that any initiative from Brussels can be free of red tape. ‘If you want to do research, then you can't lay out beforehand all the answers you're going to get,’ he says. ‘And if you try to get people to stick rigorously to a plan, then you get a lot of silly things going on. If you try to form very complex bureaucratic organisations to do the research, you get a lot of delays and so on, so things are not very timely.’

But the Framework Programme's failures need not spell disaster for the fledgling funding council, insists Lennart Philipson, former director general of the European Molecular Biology Laboratory (EMBL) in Heidelberg, Germany, and now an emeritus professor at the Karolinska Institute in Stockholm, Sweden. Drawing on his 11 years as head of EMBL, until 1993, Philipson recalls how ‘pan-European peer review was the best method for distributing the funds of EMBL and EMBO [European Molecular Biology Organization]’. The continuing high status of the two organisations, he says, is testimony that the system works. In fact, EMBO is mentioned as a possible incubator for an ERC, in spite of its specialisation.

Other proponents of the proposed research changes in the EU include 45 Nobel Laureates from Europe or of European origin, who headed a petition organised by EMBO. The European Life Scientists Organization (ELSO) organised another. Its president, Kai Simons, also the Director of the Max Planck Institute for Cell Biology and Genetics in Dresden, Germany, says research funding in Europe is just not working. ‘It's not geared for basic research—it has other aims,’ he notes. EU funds are ‘not grants, they are contracts with in-built milestones that have nothing to do [with basic research]. Basic research doesn't work like that.’

The evaluation and peer review system is falling apart, continues Simons. He says that the best people are not interested in peer reviewing a system that doesn't work: ‘You're not attracting the peer reviewers that you need to maintain quality.’ But at last, acknowledges Simons, someone in Brussels is listening. ‘In the past two years there has been enormous progress.’

## Many Questions Remain

Within a month of the Barcelona Council in 2002, the European Science Foundation (ESF), which brings together the funding agencies of 29 countries and acts as a bridge to Brussels, had formed a High Level Working Group to review the case for an ERC and how it might operate. The group, chaired by Sir Richard Sykes, Rector of Imperial College, London, United Kingdom, reported a year later, in April 2003. It endorsed the creation of an ERC as ‘the cornerstone for the ERA and the key approach to developing a locus for…long-term fundamental curiosity-driven research judged on the basis of excellence and merit’. The Sykes group also proposed, controversially, an enhanced ESF as the most effective medium for establishing an ERC swiftly.

‘Some people say that the ESF has no experience in funding large amounts… for research,’ acknowledges Enric Banda, director general of the Catalan Research Foundation in Barcelona, Spain, who finished a five-year term as the ESF's chief executive at the end of 2003 and is credited with ‘waking up’ the foundation. ‘But certainly if you create a new [organisation], that's the same thing. So the ESF is in a good position because its member organisations are the funding agencies.’

Bertil Andersson, who was a member of the Sykes Group before taking over from Banda at the ESF in January, also stakes the ESF's claim to nurture a fledgling ERC. But he accepts that any one of the respected national funding agencies, such as the German Research Foundation (DFG), or even a specialist body, such as EMBO, could do the job. ‘We don't need a new skyscraper in Brussels, but a lot of… peer review and running of the ERC could be done by existing bodies.

‘Compared to soccer, we have only the national leagues—we don't have the Champions League [the league of Europe's best teams],’ says Andersson. There is no competition for basic research grants across national boundaries in Europe, he insists. ‘The Swedish league is exciting, but the Champions League is more exciting.’

In the meantime, while the Sykes group was still deliberating, the Council of the EU appointed another group of experts to evaluate the case for an ERC. Chaired by Federico Mayor, former director general of the United Nations Educational, Scientific, and Cultural Organization (UNESCO), the ERC Expert Group also delivered its verdict—a resounding endorsement—within 12 months.

‘The first and main task for the ERC should be to support investigator-driven research of the highest quality selected through European competition,’ concluded the Mayor report, published in December 2003. ‘In doing so, the ERC should create and support nodes of excellence in European universities and research institutions, strengthening the knowledge-base that underpins economic, industrial, cultural and societal development, and thereby stimulating European competitiveness and innovative capacity at all levels.’

While few disagreed with the Mayor report's sentiments, the absence of a detailed analysis exposed underlying tensions over the rationale for an ERC. In the UK, in particular, some scientists seemed concerned that their mature and respected system for funding research risked dilution.

‘The British have always had doubts about what goes on in Europe,’ notes Kai Simons. ‘They always think that they can do it better. But the big problem for the British is that they are also too small to fund a new innovative area,’ he says. ‘Of course, we can do it without Britain, but they are an important part of Europe and it would be sad if they're not part of it.’

The agnostic John Taylor, who was a member of the Mayor group, recalls his early reservations when the group convened. ‘I'm way beyond the euphoria; I'm into practical pragmatics,’ he notes. ‘My major input into the whole thing has been to get them to “get real” instead of just philosophising. They've been using the sort of, dare I say it, Gallic approach… of thinking about the reasons why, and the philosophy, and not thinking about what you would actually do.’

Taylor dismisses the notion that wariness of the ERC is representative of a general antipathy in Britain towards European integration. ‘What we're saying is that science in the UK is not yet well-funded enough to say we would rather do this [the ERC] instead of the things that we're already trying to get done in the UK scene.’

Anticipating the Mayor report's publication, the Royal Society quickly pulled together a detailed background paper late last year that identified ‘a number of problems that need resolution, although not necessarily through the establishment of any major new institutions within Europe’. An addendum followed in March, in direct response to the Mayor report. That addendum highlighted what it saw as the paucity of solid evidence in the Mayor report and, in some cases, the confusing data in the report's case for an ERC.

On balance it looked as though the Royal Society, and as such the British science establishment as a whole, had weighed the disadvantages of an ERC as greater than its advantages, but Bob May is quick to refute this charge. ‘My vision and the Royal Society's vision of the ERC is that it will fund the very best science,’ he insists. ‘The Mayor committee itself was really good people who'd produced basically a good report…. I'm basically in favour of this European Research Council… provided it can be set up properly, which is by no means certain.’

For May, and other scientists on the continent, the ERC offers a real chance to redress the balance of fortune in favour of young scientists. ‘The way to encourage science is to get the best people and set them free to express their creativity while they are young, which means bring them into the best laboratories—don't let them get entrained in hierarchies of deference to second-rate people,’ says May.

‘The most important single thing to create one Europe in science is a flexible postdoctoral programme that gets the best young people wherever they are and lets them go to the best places,’ enthuses May. An ERC will then foster those collaborations, he forecasts. ‘It won't ask whether they're *juste retour*, whether they're serving some industrial purpose, it will just try to fund the best science. But I hope increasingly the best projects will involve collaborations, as they do in Britain, collaborations among institutions within Europe.’

##

**Box 1. Glossary of Europe**

Council of the European Union – Ruling organisation (along with European Parliament), and not to be confused with the European Council (see below). It comprises ministers from governments of the Member States, which have varying voting powers led by France, Germany, Italy, and the UK.

Euro (€) – Common European currency launched on 1 January 2002 in 12 participating Member States (the UK, Sweden, and Denmark chose to postpone adoption of the currency indefinitely).

European Commission – Executive organisation, mainly based in Brussels, run by 20 Commissioners and around 24,000 civil servants.

European Council – Body that brings together leaders of Member States to define broad policy objectives for the EU's six main institutions (Parliament, Council, Commission, Court of Justice, Court of Auditors, and Ombudsman). Meets twice a year in the Member State holding the Council's presidency, which changes every six months.

European Parliament – Elected organisation, based in Strasbourg, France, that rules the EU (jointly with the Council of the EU, see top) and will have 732 Members after the accession of the ten new Member States in May 2004.

European Research Area – Commissioner Philippe Busquin's vision for the future of research in Europe, and the main focus of the 6th Framework Programme. It aims to achieve ‘scientific excellence, improved competitiveness and innovation through the promotion of increased co-operation, greater complementarity and improved co-ordination between relevant actors, at all levels’.

European Union – Evolving political, social, and economic union of an increasing number of European countries, or Member States. First proposed in 1950 during rehabilitation after the Second World War and formally created by the Maastricht Treaty in 1992. Grew from six nations in 1951 (Belgium, France, Germany [then West Germany], Italy, Luxembourg, and the Netherlands) to nine in 1973 (addition of Denmark, Ireland, and the UK), to ten in 1981 (addition of Greece), to 12 in 1986 (addition of Spain and Portugal), to 15 in 1995 (addition of Austria, Finland, and Sweden), with a total population of 380 million people (cf. 290 million for US; 130 million for Japan). Ten more countries (Cyprus, Czech Republic, Estonia, Hungary, Latvia, Lithuania, Malta, Poland, Slovakia, and Slovenia) join in May 2004, which will lift the EU's population to 450 million people. Bulgaria and Romania are due to join in 2007, which will add another 50 million people.

Framework Programme – The EU's principal mechanism for funding research in Member States, proposed by the Commissioner for Research (Philippe Busquin) and adopted by the Council and Parliament. Framework Programmes have four-year budgets but cover five-year periods, so consecutive programmes overlap, and are prescribed two years before they begin. The 6th programme (FP6) is worth €17.5 billion (or about 4% of the EU's total budget and 5.4% of all public, nonmilitary research spending in Europe) and runs from the beginning of 2003 to the end of 2006.

*Juste retour* (fair reward) – Claim made by Member States for rewards at least equal to their share of the cost of any programme or initiative; critics say it promotes bureaucracy and uncompetitiveness.

## URLs

European Council: Presidency Conclusions - http://ue.eu.int/presid/conclusions.htm

Europa: Gateway to the European Union - http://europa.eu.int

Activities of the European Union: Research and Innovation - http://europa.eu.int/pol/rd/index_en.htm

Sixth Framework Programme - 2002–2006 - http://europa.eu.int/scadplus/leg/en/lvb/i23012

Articles about the European Research Area - http://europa.eu.int/scadplu/leg/en/lvb/i23010.htm; http://ueropa.eu.int/comm/research/era/index_en.html

## Abbreviations

ELSO: European Life Scientists Organization
EMBL: European Molecular Biology Laboratory
EMBO: European Molecular Biology Organization
ERA: European Research Area
ERC: European Research Council
ESF: European Science Foundation

## References

[pbio-0020161-Anonymous1] [Anonymous] (2004). Commission calls for boost in basic research. http://europa.eu.int/comm/research/press/2004/pr1501en.html.

[pbio-0020161-Anonymous2] [Anonymous] (2004). European Research Council must be independent: The Max Planck Society calls for decision on the establishment of a European Research Council before the end of 2004. http://www.mpg.de/english/illustrationsDocumentation/documentation/pressReleases/2004/pressRelease20040308/index.html.

[pbio-0020161-CEC1] Commission of the European Communities (2004). Communication from the Commission: Europe and basic research. http://europa.eu.int/comm/research/press/2004/pdf/acte_en_version_final_15janv_04.pdf.

[pbio-0020161-EGFM1] Expert Group chaired by Federico Mayor (2003). The European Research Council: A cornerstone in the European Research Area. http://www.ercexpertgroup.org/finalreport.asp.

[pbio-0020161-Gruss1] Gruss P (2004). Conclusions of the conference ‘Changes and Challenges for European Research Structures and Politics’ on March 1st, 2004 in Berlin. http://www.mpg.de/pdf/statementGruss.pdf.

[pbio-0020161-HLWG1] High Level Working Group chaired by Richard Sykes (2003). An ESF position paper: New structures for the support of high-quality research in Europe. http://www.esf.org/newsrelease/63/ERC.pdf.

[pbio-0020161-TRS1] The Royal Society (2004). The future funding of the European science base: A Royal Society background working paper, V1.0. http://www.royalsoc.ac.uk/templates/statements/StatementDetails.cfm?statementid=243.

[pbio-0020161-TRS2] The Royal Society (2004). The place of fundamental research in the European Research Area: The Royal Society response to the Mayor report. http://www.royalsoc.ac.uk/policy.

